# A new line tunneling SiGe/Si iTFET with control gate for leakage suppression and subthreshold swing improvement

**DOI:** 10.1186/s11671-023-03875-9

**Published:** 2023-07-28

**Authors:** Jyi-Tsong Lin, Shao-Cheng Weng

**Affiliations:** Department of Electrical Engineering, National Sun Yat-Sen University, Kaohsiung, 80424 Taiwan, ROC

**Keywords:** Control gate (CG), Tunnel FET (TFET), Subthreshold swing, Schottky contact, Line tunneling, Band-to-band tunneling (BTBT), Leakage current

## Abstract

This article presents a new line tunneling dominating metal–semiconductor contact-induced SiGe–Si tunnel field-effect transistor with control gate (CG-Line SiGe/Si iTFET). With a structure where two symmetrical control gates at the drain region are given a sufficient negative bias, the overlap of the energy bands at the drain in the OFF-state is effectively suppressed, thus reducing the tunneling probability and significantly decreasing leakage current. Additionally, the large overlap area between the source and gate improves the gate’s ability to control the tunneling interface effectively, improving the ON-state current and subthreshold swing characteristics. By using the Schottky contact characteristics of a metal–semiconductor contact with different work functions to form a PN junction, the need to control doping profiles or random doping fluctuations is avoided. Furthermore, as ion implantation is not required, issues related to subsequent annealing are also eliminated, greatly reducing thermal budget. Due to the different material bandgap characteristics selected for the source and drain regions, the probability of overlap of the energy bands in the source region in the ON-state is increased and that in the drain region in the OFF-state is reduced. Based on the feasibility of the actual fabrication process and through rigorous 2D simulation studies, improvements in subthreshold swing and high on/off current ratio can be achieved simultaneously based on the proposed device structure. Additionally, the presence of the control gate structure effectively suppresses leakage current, further enhancing its potential for low-power-consumption applications.

## Introduction

With the flourishing development of the Internet of Things (IoT) and wearable devices, MOSFETs must continue to be scaled down in order to enhance their operating efficiency and switching speed. However, as MOSFETs rapidly decrease in size, some devices suffer from high standby and dynamic power consumption [1]. This poses a significant challenge in achieving energy efficient goals, and thus it is necessary to reduce leakage current and operating voltage while maintaining a steep subthreshold swing (SS) to achieve sufficient On-state current (*I*_ON_) to drive the circuits. However, the current conduction mechanism of conventional MOSFETs is limited by thermal restrictions, which makes the subthreshold swing unable to go below 60 mV/dec at room temperature. To make the subthreshold swing not affected by thermal limitations, the Band-to-Band Tunneling (BTBT) mechanism [2] that is not subject to thermal restrictions must be used. TFETs use the BTBT mechanism to achieve an SS smaller than 60 mV/dec [3, 4]. TFETs have shown some promising features, such as low SS, low power consumption, lower leakage current, and the ability to suppress the Short Channel Effects (SCEs) that may occur during MOSFET scaling [5, 6]. Moreover, compared to the diffusion and drift mechanisms used for MOSFET conduction, the BTBT mechanism is less sensitive to temperature, making TFET a promising candidate to replace MOSFETs. However, TFETs still have the disadvantage of lower *I*_ON_ compared to MOSFET. Conventional TFET structure is similar to MOSFET [7] but differs in that the source and drain of MOSFET have the same doping type, while those of TFET have different doping types. A common TFET is *P*–*I*–*N* structure. Nonetheless, the conventional TFET fabrication process requires three implantations, and high-temperature annealing is required after implantation. As semiconductor device sizes continue to scale, process challenges arise from increased thermal budget and more difficult to control doping profiles and interface material quality, which further exacerbates the trap-assisted tunneling (TAT) effect [8] and deteriorates the subthreshold swing characteristics. The conventional TFET faces three challenges: (1) TAT effect; (2) high thermal budget; and (3) low ON-state current. To overcome these limitations, various solutions have been proposed in many papers, such as heavily doped Gaussian drain regions [9], multiple gates [10–13], pocket structures [14], heterostructures formed using small bandgap materials [15–19], the high electron mobility characteristics of III–V materials [20, 21], ferroelectric material NC-TFET [22], or more novel two-dimensional materials [23, 24].

**Fig. 1:**
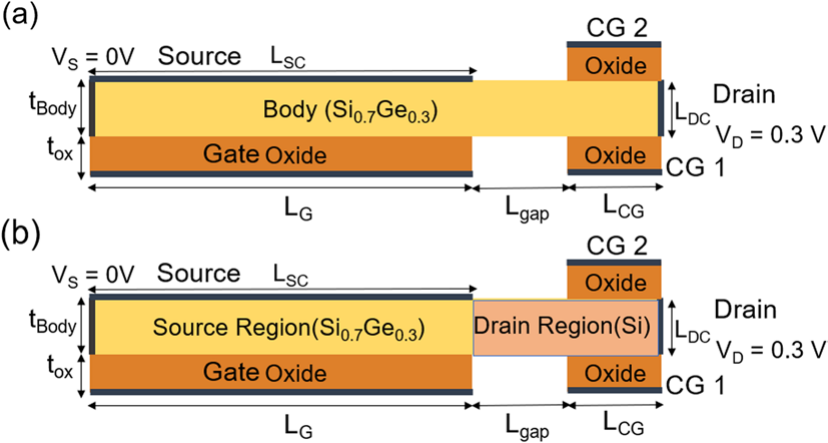
Schematic cross-sectional view of **a** CG-Line iTFET and **b** CG-Line SiGe/Si iTFET

The tunneling current generation mechanism of TFET can be divided into two types: “point tunneling” and “line tunneling” [25]. In the conventional TFET, a gate controls a PIN diode based on the BTBT mechanism under reverse bias, and tunneling phenomenon occurs at the junction of the source and channel. This results in a limited tunneling area, known as point tunneling. In contrast, another type of tunneling mechanism, known as line tunneling, produces a much large tunneling area and sharper doping profile distribution through gate-source overlapping, using a gate to control a PN diode based on the BTBT mechanism under reverse bias. To improve our device characteristics, the gate and source are overlapped completely to produce the largest possible line tunneling area and suppress point tunneling, thereby improving *I*_ON_ and SS. Although TFETs with a gate-source overlapped structure as the conduction mechanism have better *I*_ON_ and SS, they still require different doped regions for PN junctions. To reduce the thermal budget caused by the number of implantation and annealing processes required by the conventional structure, we propose the use of bulk materials with the same material for the source and drain, and metal contacts for the source and drain, which generate Schottky and Ohmic contacts due to their different metal work functions. By using an *M*–*S* interface to replace the original *P*–*N* junction [26], thereby reducing the number of required implantation process and greatly reducing the heat budget. Conventional TFETs are based on Si substrates that have a larger and indirect bandgap. This limits the likelihood of BTBT occurring, resulting in lower *I*_ON_ and SS below 60 mV/dec only in the low current range [27]. To address the issue of low *I*_ON_, we use SiGe material with a composition of 70% Si and 30% Ge as the substrate in our device, which has a smaller bandgap and can increase the probability of BTBT, leading to a significant improvement in the *I*_ON_ and *I*_ON_/*I*_OFF_ ratio, allowing for a reduction in *V*_DD_ and meeting the for low-power-consumption requirements of device applications. In addition, SiGe materials are all Group IV elements, which enhances their compatibility with Si-CMOS processes. Next, we use heterostructures with different body materials to improve the source region that affects the ON-state and the drain region that affects the OFF-state. By changing the material and body doping concentration, we are able to further reduce the leakage current in the OFF-state, achieving *I*_ON_/*I*_OFF_ = $1.22 \times {10^{9}}$ and SS_avg_ = 19.12 mV/decade with a gate length of 50 nm. This presents an opportunity to replace MOSFETs and meet the demands of low power consumption and energy efficient in future applications. This article consists of the following sections. In “Device Design and Simulation Method” Section, we introduce the device design (Fig. 1 and Table 1) and simulation method. In “Electrical characteristics discussion” Section, we discuss the effects of the device’s electrical characteristics under various parameter variations and simulation results. Finally, “Conclusion” Section concludes the achievements.

**Table 1:** Device parameters

Parameter	Value
Gate oxide thickness (*t*_ox_)	3 nm
Gate length (*L*_G_)	50 nm
Body thickness (*t*_Body_)	5 nm
Control gate length (*L*_CG_)	10 nm
Body doping(*n*-type) in CG-Line iTFET	1 × 10^19^ cm^−3^
Doping of source region (*n*-type) in CG-line SiGe/Si iTFET	1 × 10^17^ cm^−3^
Doping of drain region (n-type) in CG-line SiGe/Si iTFET	1 × 10^19^ cm^−3^
Drain metal contact length (*L*_DC_)	5 nm
Source metal contact length (*L*_SC_)	50 nm
Gate-control gate gap (*L*_gap_)	10 nm

## Device design and simulation method

**Fig. 2:**
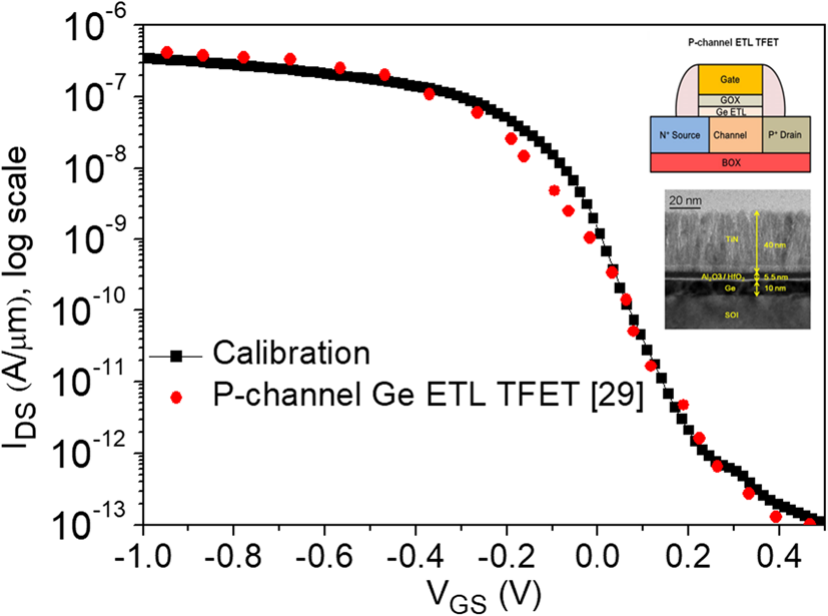
TCAD model calibration using experimental data [29]

In this article, we use Sentaurus TCAD [28] to simulate the electrical characteristics of our proposed CG-Line iTFET structure under various parameter variations. To calculate tunneling current, nonlocal charge carrier tunneling model is employed. To properly calculate the impact of non-ideal device characteristics, we use Shockley–Read–Hall (SRH) models. Due to the thin channel thickness, it is necessary to consider the quantum confinement effect. To ensure the accuracy and feasibility of our simulations, we have calibrated our simulation model using an actual fabricated Ge ETL TFET [29], as shown in Fig. 2. Equation (1) represents the calculation formula for tunneling generation rate, and the parameter adjustments during the calibration process are shown in Table 2. Figure 1 shows the CG-Line iTFET and CG-Line SiGe/Si

1 $$G = A\left( {\frac{F}{{F_{O} }}} \right)^{P} {\text{exp}}\left( { - \frac{B}{F}} \right)$$

**Fig. 3:**
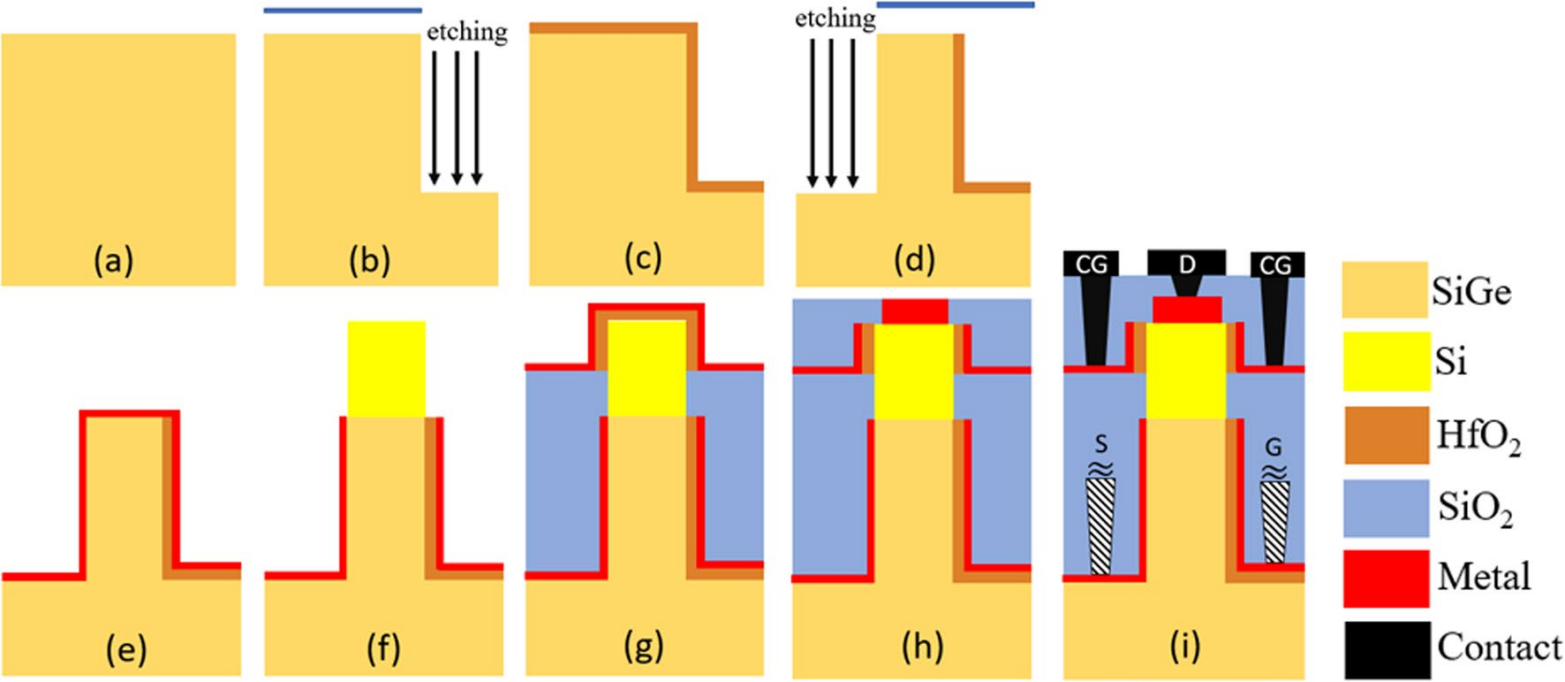
**a–i** Fabrication process steps of CG-Line SiGe/Si iTFET

iTFET structures, and various device parameters are presented in Table 1. The gate length (*L*_G_) is 50 nm. The source metal contact length (*L*_SC_) is 50 nm with an appropriate metal work function selected for a Schottky contact with a barrier height 0.8 eV to the semiconductor. The drain metal length is 5 nm and is in ohmic contact with the semiconductor. The gate and control gate (CG) oxide materials are hafnium oxide (HfO_2_) with a thickness of 3 nm. The work function of the gate electrode is 3.7 eV. The length of the control gate (*L*_CG_) is 10 nm. The body of the CG-Line iTFET is made of Si_0.7_Ge_0.3_ with a thickness of 5 nm and is doped with *N*-type phosphorus (10^19^ cm^−3^). The body of CG-Line SiGe/Si iTFET is divided into source and drain regions. The source region material is Si_0.7_Ge_0.3_ doped with *N*-type phosphorus (10^17^ cm^−3^), and the drain region material is Si doped with *N*-type phosphorus (10^19^ cm^−3^). In Fig. 3, we present the fabrication steps of CG-Line SiGe/Si iTFET. In Fig. 3a, an *N*-type doped SiGe substrate is shown. Etching is performed using a mask, followed by the deposition of HfO_2_ using ALD, as shown in Fig. 3b–c. The upper part of the HfO_2_ is then removed, and selective etching is carried out, as depicted in Fig. 3d. Subsequently, source and gate metal are deposited, and the upper metal layer is removed, followed by selective epitaxial growth of Si. Then, in situ doping is performed, as shown in Fig. 3e–f. Next, deposit SiO_2_, followed by the deposition of HfO_2_ as the oxide layer for the Control Gate (CG). Then, deposit metal and remove the upper portion, as shown in Fig. 3g. Subsequently, deposit SiO_2_ and selectively etch SiO_2_, followed by the deposition of Drain metal, as shown in Fig. 3h. Finally, deposit TEOS and establish electrode-to-metal contact, as shown in Fig. 3i. It should be noted that the metal contact for the source and gate is made in the front, while the CG and Drain are connected at the back. During subsequent electrical characterization, the OFF-state current (*I*_OFF_) is the current at the minimum subthreshold swing, and the voltage *V*_OFF_ is the moment when tunneling current is initiated. The *I*_ON_ calculation is performed for the current at *V*_G_ = *V*_OFF_ + 0.5 V. The threshold voltage (*V*_th_) is determined by the current–voltage curve at a constant current of $1 \times 10^{ - 7}$ A, which is commonly referred to as the constant current method [30]. The average subthreshold swing (SS_avg_) is given by SS_avg_ = (*V*_th_ − *V*_OFF_)/(log(*I*_th_) − log(*I*_OFF_)).

**Table 2:** Calibration parameters [31]

Parameters	Si_*x*_Ge_1*−x*_(*x* = 1)	Si_*x*_Ge_1−*x*_(*x* = 0)
*µ*_**n**_	1414 cm^2^/(V s)	3900cm^2^/(V s)
*µ*_**p**_	470 cm^2^/(V s)	1900cm^2^/(V s)
*A*	4 × 10^14^ cm^−3^ s^−1^	9.1 × 10^16^ cm^−3^ s^−1^
*B*	1.9 × 10^7^ V cm^−1^	4.9 × 10^6^ V cm^−1^
*P*	2.5	2.5
*F*_**0**_	1 V/m	1 V/m

## Electrical characteristics discussion

### Effects of variations in control gate structure and bias voltage

**Fig. 4:**
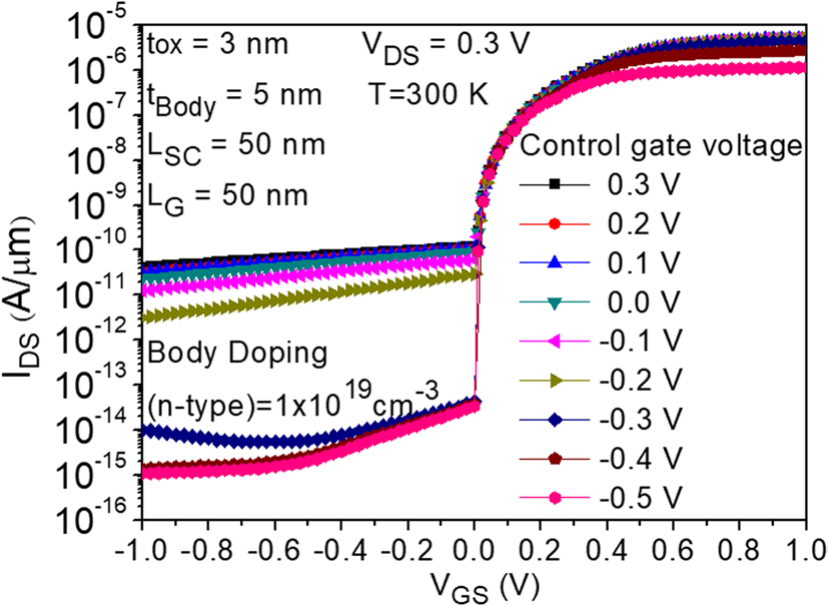
Impact of control gate voltage (*V*_CG_) on the transfer characteristics of CG-Line iTFET

**Fig. 5:**
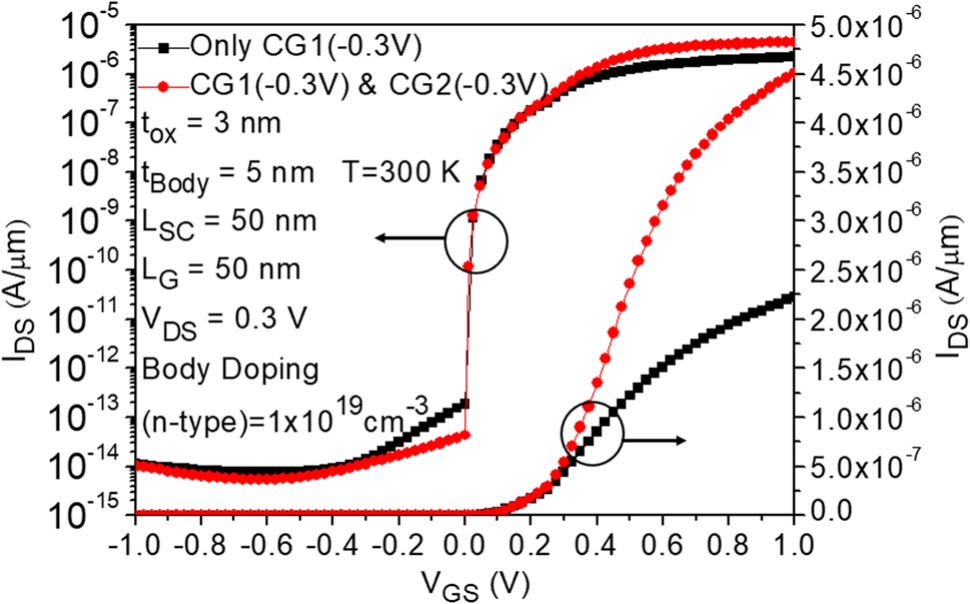
Comparison of transfer characteristics in CG-Line iTFET structures with one control gate (CG1) or with two control gates (CG1 and CG2)

**Fig. 6:**
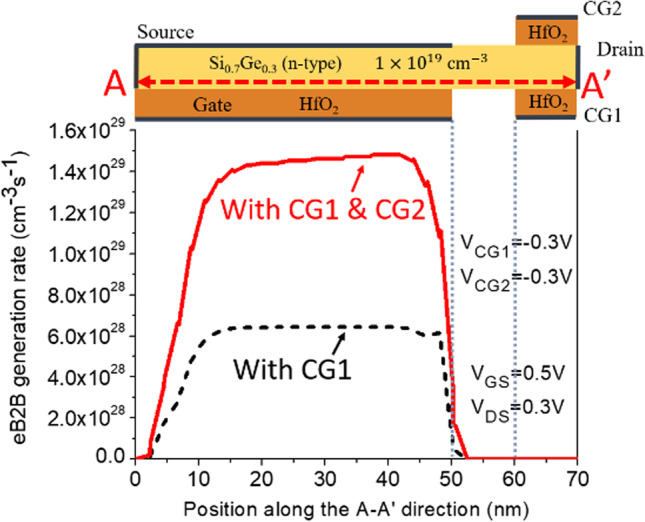
Comparison of electron BTBT generation rate in CG-Line iTFET structures with one control gate (CG1) or with two control gates (CG1 and CG2) at *V*_GS_ = 0.5 V and *V*_DS_ = 0.3 V

**Fig. 7:**
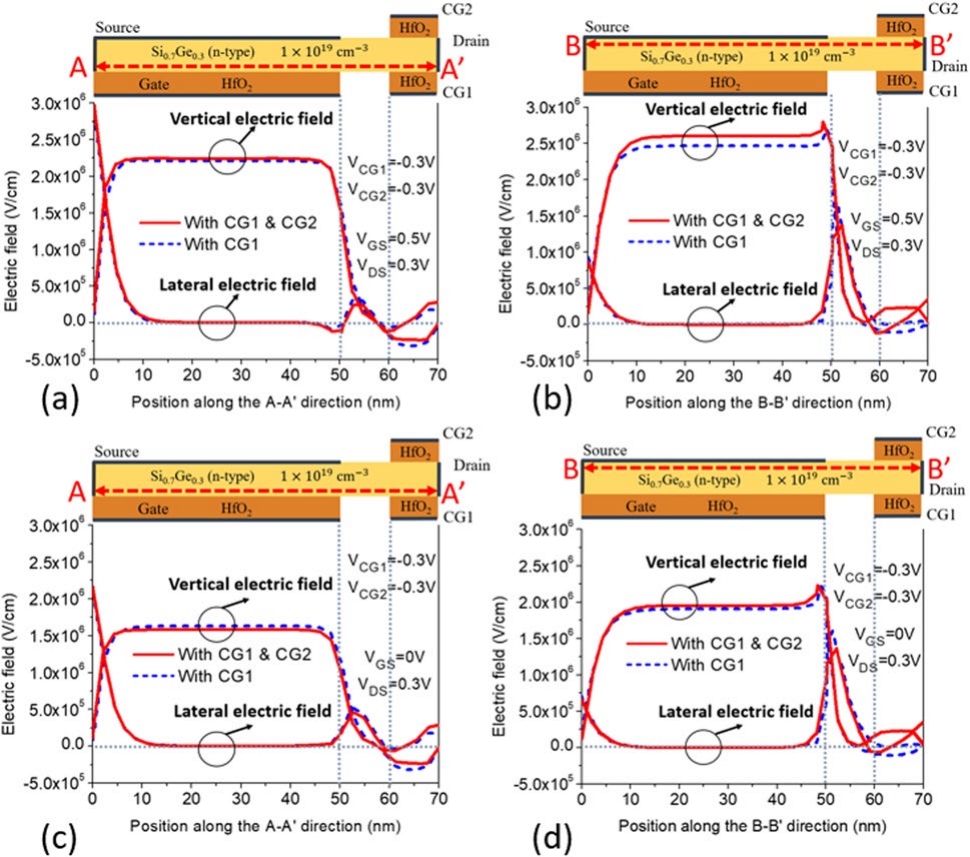
Comparison of vertical and lateral electric field in CG-Line iTFET structures with one control gate (CG1) or with two control gates (CG1 and CG2) along **a** *A*–*A*′ cutline at *V*_GS_ = 0.5 V and *V*_DS_ = 0.3 V. **b** *B*–*B*′ cutline at* V*_GS_ = 0.5 V and *V*_DS_ = 0.3 V. **c** *A*–*A*′ cutline at *V*_GS_ = 0 V and *V*_DS_ = 0.3 V. **d** *B*–*B*′ cutline at *V*_GS_ = 0 V and *V*_DS_ = 0.3 V

**Fig. 8:**
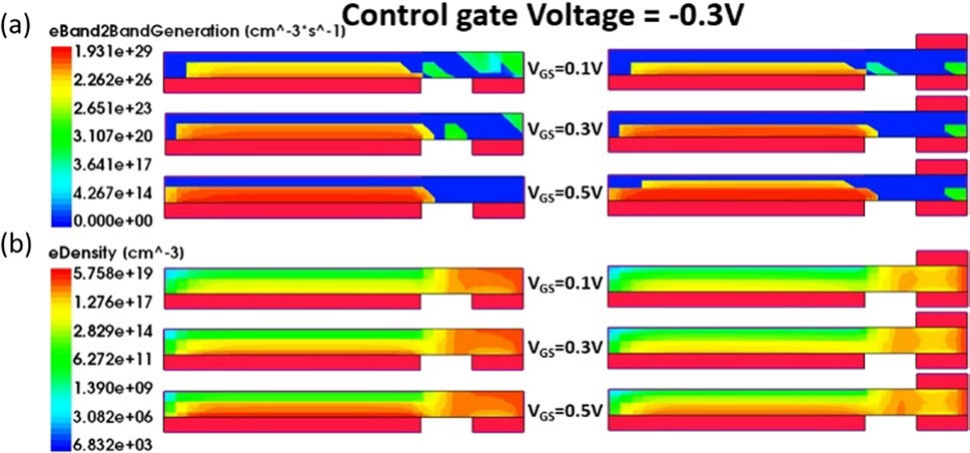
Comparison of **a** Electron BTBT generation rate. **b** Electron density in CG-Line iTFET structures with one control gate (CG1) or with two control gates (CG1 and CG2) at *V*_DS_ = 0.3 V and different *V*_GS_

Figure 1a depicts the structure of CG-Line iTFET, in which both above and below the drain side of the body are equipped with additional control gates, namely Control Gate 1 (CG1) and Control Gate 2 (CG2). The lengths of CG1 and CG2 are both 10 nm, and they are spaced equidistant from *L*_gap_. Figure 4 illustrates the impact of changing the bias voltage of the control gates on the transfer characteristics curve. The bias voltages of CG1 and CG2 are kept the same, ranging from 0.3 to − 0.5 V. As the voltage gradually decreases from 0.3 to − 0.5 V, the OFF-state current decreases accordingly until it becomes less noticeable at − 0.3 V. Although significant suppression of the ambipolar effect occurs at − 0.4 V and − 0.5 V for the control gate voltages, the ON-state current also decreases, which reduces the *I*_ON_/*I*_OFF_ current ratio. Therefore, a preferable option is to use − 0.3 V as the bias voltage for both CG1 and CG2. Figure 5 compares the transfer characteristics curves of structures with only CG1 and structures with both CG1 and CG2, where the bias voltage of both control gates is − 0.3 V. As shown in Fig. 5, the structure with control gates above and below the drain side exhibits better ON-state and OFF-state current characteristics compared to the structure with only CG1. The ON-state current is closely related to the BTBT generation rate in the source and gate overlapping region, as shown in Fig. 6, and the *A*–*A′* cutline represents the region near the gate oxide layer at 1 nm, with a gate voltage of 0.5 V and a fixed bias voltage of − 0.3 V for the control gates. As shown in Fig. 6, the BTBT generation rate is more than doubled in the structure with two control gates compared to the structure with only one control gate, resulting in an improvement in the ON-state current. This is also demonstrated in Fig. 5. The enhancement of tunneling through the gate and source regions is related to the electric field. This electric field can be separated into vertical and lateral components, which affect line and point tunneling, respectively. To simplify the analysis, the electric field direction is reversed by multiplying it by − 1. This does not affect the magnitude comparison. In Fig. 7a, with a gate voltage of 0.5 V in the ON-state, the *A*–*A*′ cutline is located near the gate oxide layer at 1 nm. It can be observed from the graph that in the vertical electric field, the structure with two control gates exhibits a stronger electric field than the structure with only one control gate. This can enhance the ability of line tunneling and increase the ON-state current. In the lateral electric field, the structure with two control gates exhibits a weaker electric field than the structure with only one control gate. This reduces the occurrence of point tunneling near the drain region. This is consistent with our goal of having line tunneling dominate and suppressing point tunneling. The difference between the vertical and lateral electric fields in different structures is more apparent in the *B*–*B*′ cutline near the source metal at 1 nm, as shown in Fig. 7b. In the graph, it can be observed that the structure with two control gates exhibits enhanced vertical electric field and weakened lateral electric field. When the structure includes two control gates, the symmetric fixed negative bias voltage causes the lateral electric field generated by the drain voltage to diverge between the right side of the source contact metal and the drain region. The electric field in the upper half of the region near the drain, which originally had a larger influence range, becomes concentrated in the middle area. Consequently, compared to a structure with only one control gate, a significant reduction in the intensity of the lateral electric field can be observed at the *B*–*B*′ cutline. However, both structures have control gate (CG1) beneath the drain region, resulting in a less pronounced decrease in the lateral electric field intensity at the *A*–*A*′ cutline. Figure 7c, d compares the vertical and lateral electric fields at a gate voltage of 0 V in the OFF-state. In Fig. 7c, it can be seen that the vertical electric field is smaller in the structure with two control gates at the position of the *A*–*A*′ cutline compared to the structure with only one control gate, but its impact is not significant in the OFF-state. However, the lateral electric field is still reduced in the structure with two control gates, which can suppress point tunneling and reduce leakage current in the OFF-state, which is desirable. Figure 7d corresponds to the position of the *B*–*B*′ cutline, where the increase in vertical electric field in the structure with two control gates is not significant. However, the lateral electric field still has a suppressive effect, reducing the probability of tunneling and leakage current in the OFF-state. Low leakage current in the OFF-state is important for low-power-consumption devices. In addition, in Fig. 5, we can observe that the ON-state current in the two structures with different control gates are almost the same before the gate voltage reaches 0.3 V. However, beyond 0.3 V, it can be clearly seen that the structure with two control gates exhibit a significant increase in current. From Fig. 8a, it is evident that the BTBT generation rate in the structure with two control gates significantly increase compared to that in the structure with only one control gate when the gate voltage is above 0.3 V. One possible reason for this is apparent from Fig. 8b, where it can be seen that the carrier concentration accumulation near the body region, close to the gate, is slower in the structure with two control gates after the gate voltage reaches 0.3 V. Therefore, when the tunneling junction is fully open, the shielding effect caused by the carrier accumulation in the channel region is reduced, thereby enhancing the gate control capability. Furthermore, in Fig. 8b, it is observed that as the gate voltage increases, both the electron accumulation layer concentration and region expand. Consequently, the *P*-type region at the semiconductor surface on the right side of the gate gradually diminishes. This diminishing P-type region leads to the disappearance of the *P*–*N* junction for point tunneling; as a result, the green patches near the drain, as shown in Fig. 8a, gradually disappear.

**Fig. 9:**
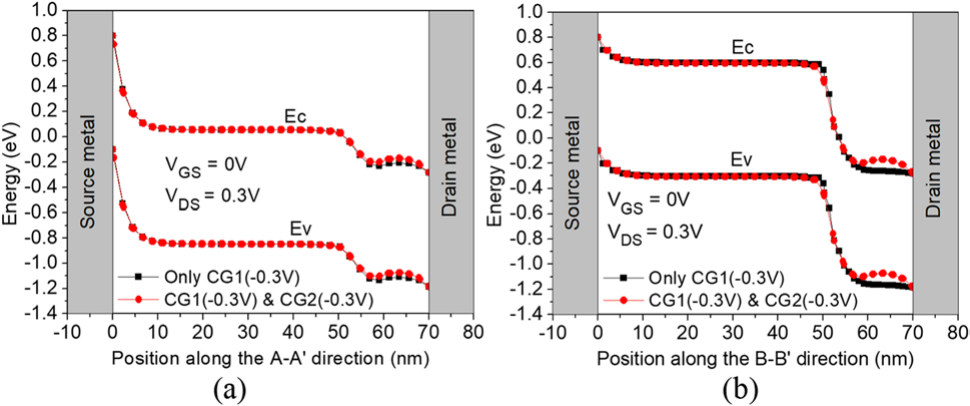
Comparison of energy band diagram in CG-Line iTFET structures with one control gate (CG1) or with two control gates (CG1 and CG2) along **a** *A*–*A*′ cutline and **b** *B*–*B*′ cutline at *V*_GS_ = 0 V and *V*_DS_ = 0.3 V

Figure 9 illustrates the effect of the two control gate structures on the energy bands in the OFF-state. In Fig. 9a, it can be observed that the energy bands do not overlap significantly. However, in the structure with two control gates (control gate 1 and control gate 2) as shown in Fig. 9b, the tunneling distance is longer near the drain region compared to the structure with only control gate 1. The cutline in the lateral direction represents the point tunneling mechanism, which can mitigate the impact of point tunneling in the OFF-state and consequently reduce leakage current.

### Effects of mole fraction variations in Si_***x***_Ge_1−***x***_ materials

**Fig. 10:**
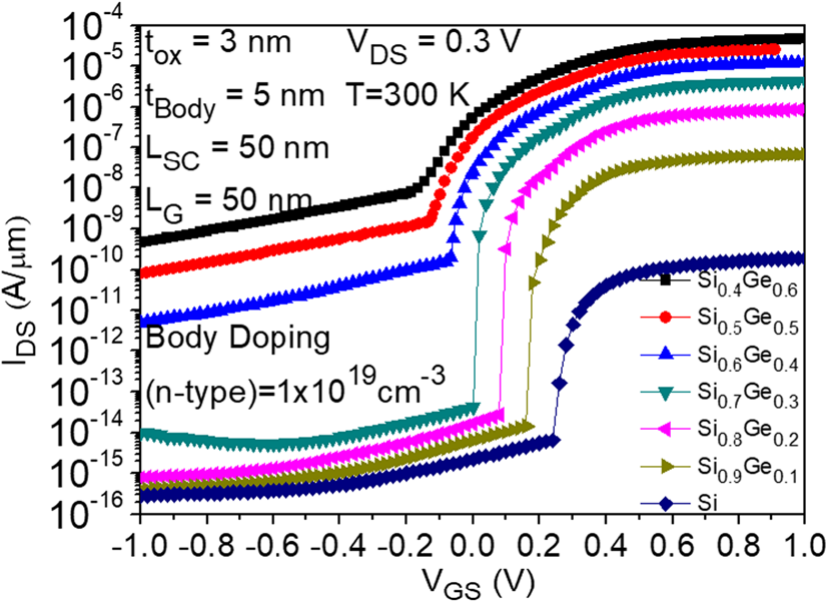
Impact of Si_*x*_Ge_1−*x*_ mole fraction of body material on the transfer characteristics of CG-Line iTFET

Figure 10 compares the impact of changing the mole fraction of Si_*x*_Ge_1−*x*_ in the body material on the device performance. The device structure has two control gates at the drain end with a bias of − 0.3 V, as shown in Fig. 1a. The wider energy gap of Si reduces the likelihood of band overlap and thus decreases tunneling probability compared to Ge, which has a narrower energy gap. As the mole fraction of Ge increases, the Si content decreases, resulting in a lower energy gap of Si_*x*_Ge_1−*x*_. From the graph, it can be observed that using Si as the body material results in lower leakage current compared to other Si_x_Ge_1-x_ compositions, due to its wider energy gap. However, the ON-state current also decreases, which is not desired. On the other hand, Si_0.4_Ge_0.6_, Si_0.5_Ge_0.5_, and Si_0.6_Ge_0.4_, with higher Ge content, exhibit larger ON-state currents due to their smaller energy gaps, which make tunneling more likely. However, the leakage current in the OFF-state also increases. Finally, Si_0.7_Ge_0.3_ is selected as the body material, as it has higher ON-state current and a larger* I*_ON_/*I*_OFF_ ratio compared to Si_0.8_Ge_0.2_.

### Effects of source and drain body material variations

**Fig. 11:**
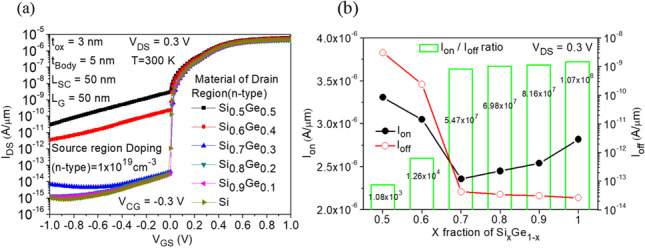
**a** Impact of body material of drain region on the transfer characteristics of CG-Line SiGe/Si iTFET. **b** *I*_ON_, *I*_OFF_ and* I*_ON_/*I*_OFF_ ratio with different mole fraction of Si_*x*_Ge_1−*x*_ of drain region

To achieve the goal of increasing the ON-state current and reducing the leakage current, we can use materials with narrow bandgaps to increase tunneling probability. As mentioned above, we modified the entire body material, and although the narrow bandgap leads to easier overlapping of the ON-state energy bands, thereby increasing the current, the ON-state is primarily dominated by tunneling in the source region, while leakage current is dominated by tunneling in the drain region. While narrow bandgap materials can improve the energy band overlapping in the source region during the ON-state, they also increase the probability of tunneling and leakage current during the OFF-state. Therefore, we attempt to use different materials to investigate their effects. We primarily used narrow bandgap materials in the source region and wider bandgap materials in the drain region, as shown in Fig. 1b. In Fig. 11a, the source region material is Si_0.7_Ge_0.3_, while varying the mole fraction of Si_*x*_Ge_1−*x*_ in the drain region. It can be observed from the graph that increasing the Si content in the drain region material reduces the leakage current, as the wider bandgap reduces the band overlap and tunneling probability in the OFF-state drain region. According to Table 3, the bandgap of Si_*x*_Ge_1−*x*_ decreases as the Ge content increases compared to pure Si (*x* = 1). SiGe has a smaller bandgap and effective carrier mass than Si, which results in lower tunneling barriers. From Fig. 11b, it can be observed that although the ON-state current tends to decrease and then increase with increasing Si mole fraction in the Si_*x*_Ge_1−*x*_ drain region, the change is not significant. However, using Si in the drain region significantly decreases the OFF-state leakage current, thus achieving the highest *I*_ON_/*I*_OFF_.

**Table 3:** Band gap of sige with different mole fraction [31]

	Band gap (eV)
Si	1.12
Si_0.9_Ge_0.1_	1.074
Si_0.8_Ge_0.2_	1.028
Si_0.7_Ge_0.3_	0.982
Si_0.6_Ge_0.4_	0.936
Si_0.5_Ge_0.5_	0.89

### Effects of source and drain body material doping concentration variations

**Fig. 12:**
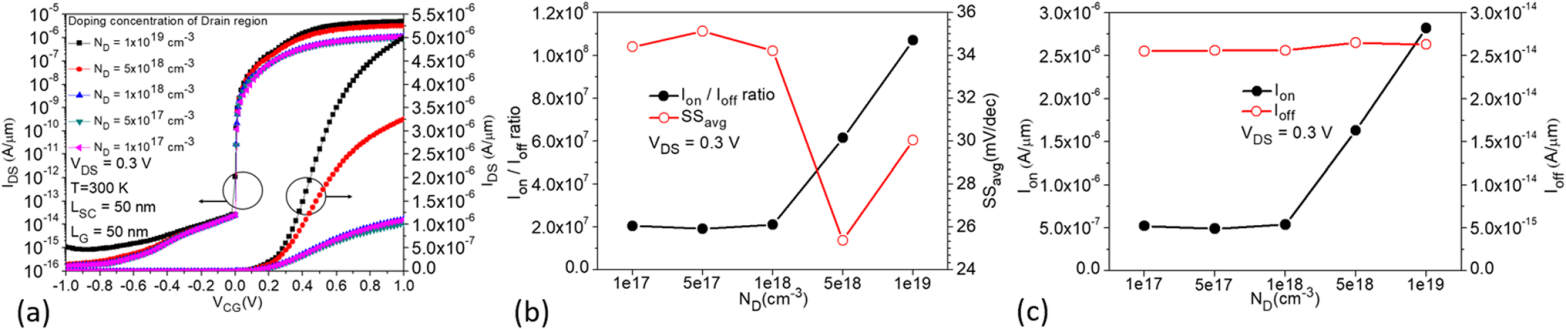
**a** Impact of doping concentration of Drain region on the transfer characteristics of CG-Line SiGe/Si iTFET. **b** *I*_ON_/*I*_OFF_, SS_avg_, **c** *I*_ON,_ and *I*_OFF_ with different doping concentration (*N*_D_) of drain region

**Fig. 13:**
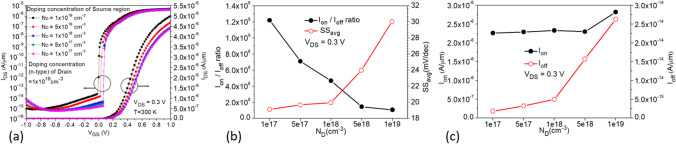
**a** Impact of doping concentration of source region on the transfer characteristics of CG-Line SiGe/Si iTFET. **b** *I*_ON_/*I*_OFF_, SS_avg_, **c** *I*_ON,_ and *I*_OFF_ with different doping concentration (*N*_D_) of source region

**Fig. 14:**
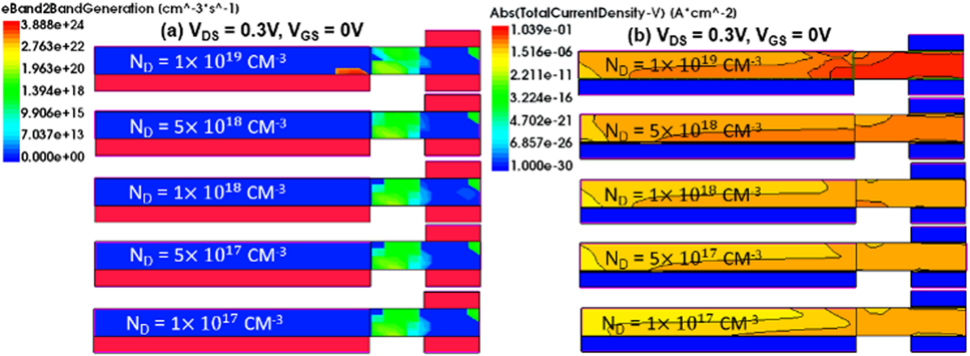
Comparison of **a** Electron BTBT generation rate. **b** Total current density in CG-Line SiGe/Si iTFET structures under varying source region doping concentration (*N*_D_)

**Fig. 15:**
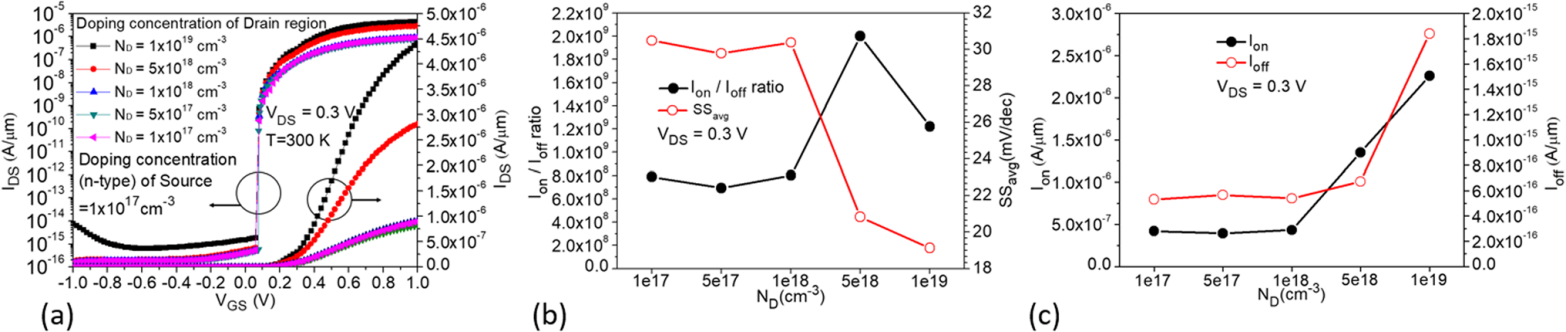
**a** Impact of doping concentration of Drain region on the transfer characteristics of CG-Line SiGe/Si iTFET. **b** *I*_ON_/*I*_OFF_, SS_avg_, **c** *I*_ON,_ and *I*_OFF_ with different doping concentration (N_D_) of Drain region

In Fig. 12a, the source region is Si_0.7_Ge_0.3_ with n-type doping of $1 \times 10^{19}$ cm^−3^, while the drain region is Si with the same n-type doping, as shown in Fig. 1b. When the doping concentration in the drain region is decreased from $1 \times 10^{19}$ to $1 \times 10^{17}$ cm^−3^, transfer characteristic curves indicate a significant decrease in the ON-state current, while the leakage current and ambipolar effect are noticeably suppressed. However, there is no significant change observed at *V*_GS_ = 0 V. From the comparison of various characteristic parameters in Fig. 12b, c, it is evident that although the average subthreshold swing (SS_avg_) at $5 \times 10^{18}$ cm^−3^ doping concentration in the drain region is better than that at $1 \times 10^{19}$ cm^−3^, both *I*_ON_ and *I*_ON_/*I*_OFF_ decrease. Therefore, we choose a doping concentration of $1 \times 10^{19}$ cm^−3^ in the drain region. Next, with the doping concentration in the drain region fixed at $1 \times 10^{19}$ cm^−3^, we decrease the doping concentration in the source region from $1 \times 10^{19}$ cm^−3^ to $1 \times 10^{17}$ cm^−3^, as shown in Fig. 13a. It is observed that as the concentration decreases, the ON-state current also decreases, but not significantly, as shown in Fig. 13c. However, a significant suppression and decrease of leakage current can be observed when the doping concentration in the source region is $1 \times 10^{17}$ cm^−3^, resulting in better *I*_ON_/*I*_OFF_ ratio and SS_avg_ characteristics, as shown in Fig. 13b. The comparison plot of the BTBT generation rate in Fig. 14a shows that a small portion of higher concentration line tunneling occurs near the source-drain region when the source doping concentration is $1 \times 10^{19}$ cm^−3^, resulting in the formation of a parasitic transistor in the OFF-state and a significant increase in *I*_OFF_. The OFF-state current decreases as the source doping concentration decreases, as seen in the current density plot in Fig. 14b, where a higher current density is observed near the drain region when the source doping concentration is $1 \times 10^{19}$ cm^−3^. The best performance improvement is achieved when the source doping concentration is fixed at $1 \times 10^{17}$ cm^−3^ and the drain doping concentration is varied, as shown in Fig. 15a. When the drain doping concentration is decreased from $1 \times 10^{19}$ to $5 \times 10^{18}$ cm^−3^, the OFF-state current significantly decreases, but *I*_ON_ also decreases accordingly, as shown in Fig. 15c. Although there is a higher* I*_ON_/*I*_OFF_ when the drain doping concentration is $5 \times 10^{18}$ cm^−3^, the SS_avg_ characteristics show a slight degradation and *I*_ON_ also decreases significantly, as shown in Fig. 15b. Therefore, the doping concentration in the drain region is still chosen as $1 \times 10^{19}$ cm^−3^, considering the effects of the variations in source and drain doping concentrations. Based on the effects of source and drain doping concentration variations, we observed that the changes in the ON-state current are less pronounced when the source doping concentration is varied with a fixed drain doping concentration, whereas changes are more significant when the drain doping concentration is varied with a fixed source doping concentration.

### Effects of trap-assisted tunneling (TAT)

**Fig. 16:**
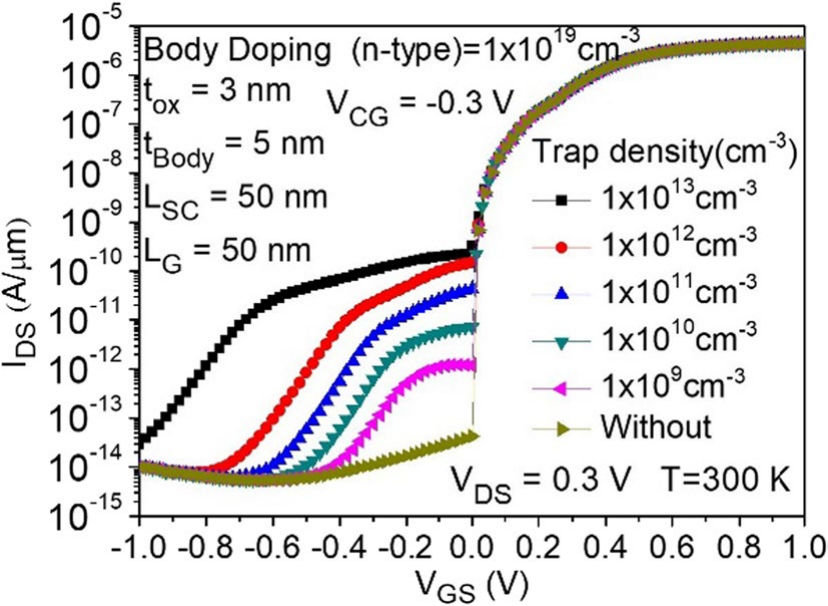
Impact of interface traps on the transfer characteristics of CG-Line iTFET

In Fig. 16, we observe the impact of interface trap density variations on the characteristics of the CG-Line iTFET. As the trap density gradually increases, it significantly affects the device behavior in the OFF-state, where point tunneling dominates. With the increase in trap density, the leakage current gradually rises. In comparison with the conventional gated pin TFET, our proposed iTFET demonstrates higher immunity to trap-assisted tunneling (TAT) due to the line tunneling mechanism. This feature ensures that our device is no longer affected by TAT in the ON-state.

**Fig. 17:**
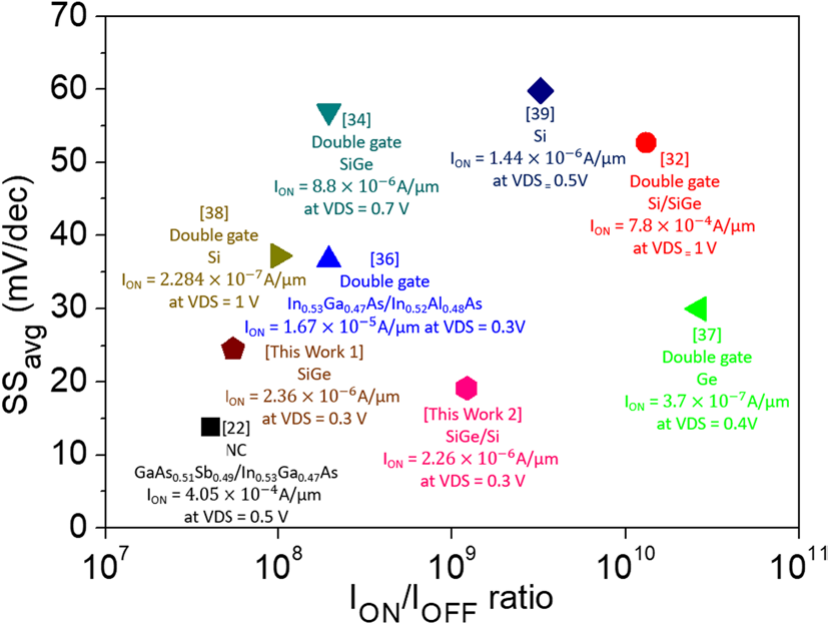
Benchmark of TFETs with various structures and materials

In Table 4, we compare our device with other Si, Ge, or SiGe-based devices. After optimizing the Work 1 to the Work 2, there is a significant improvement in both the SS_avg_ and *I*_ON_/*I*_OFF_ characteristics. Although *I*_ON_ is somewhat lower than that of other TFETs reported in the literature, V_DS_ is the lowest among them. In Fig. 17, we compare our device with other heterojunction or ferroelectric material negative capacitance and double gate TFETs [22, 32, 34, 36–39].

**Table 4:** Performance comparison of some Si and Ge TFET architectures

	Structure	Material	*I*_ON_ (A/μm)	SS_avg_ (mV/dec)	*I*_ON_/*I*_OFF_
Ref [32]. (2019)	DLG-TFET	Si	$2.84 \times 10^{ - 5}$(*V*_DS_ = 2 V)	55.3	$3.57 \times 10^{11}$
Ref [33]. (2019)	ADMDG-TFET	Si/SiGe	$7.80 \times 10^{ - 4}$(*V*_DS_ = 1 V)	52.7	$1.32 \times 10^{10}$
Ref [34]. (2021)	CPSIG-TFET	SiGe/Si	$8.8 \times 10^{ - 6}$(*V*_DS_ = 0.7 V)	57	$1.96 \times 10^{8}$
Ref [35]. (2022)	vertical CDL-TFET	Ge–Si	$3 \times 10^{ - 5}$(*V*_DS_ = 0.7 V)	34.39	$1.57 \times 10^{11}$
This work 1	CG-Line iTFET	SiGe	$2.36 \times 10^{ - 6}$(*V*_DS_ = 0.3 V)	24.48	$5.47 \times 10^{7}$
This work 2	CG-Line SiGe/Si iTFET	SiGe/Si	$2.26 \times 10^{ - 6}$(*V*_DS_ = 0.3 V)	19.12	$1.22 \times 10^{9}$

## Conclusion

In this article, based on the feasibility of the actual fabrication process, a new line tunneling dominating metal–semiconductor contact-induced SiGe/Si tunnel field-effect transistor with control gate (CG-Line iTFET) has been presented. Using the large overlap between the source and gate regions to create line tunneling can enhance *I*_ON_ and SS characteristics. The metal work function was properly adjusted to form a Schottky contact at the metal–semiconductor interface and induce carrier concentration of opposite type, avoiding issues associated with controlling doping profiles or random doping fluctuations. By reducing the number of ion implantation steps, the need for subsequent annealing repairs is avoided, leading to significant cost savings and a greatly reduced thermal budget. Due to the use of the Control gates, the ON-state line tunneling capability can be effectively improved and the OFF-state point tunneling suppressed under sufficient negative bias of Control gates, resulting in a significant enhancement of the SS. Based on the feasibility of the actual fabrication process and through rigorous 2D simulation studies, the CG-Line SiGe iTFET structure with dual control gates, when a fixed bias of − 0.3 V was applied, the device exhibited *I*_ON_ = $2.36 \times 10^{ - 6}$ A/μm, *I*_OFF_ = $4.31 \times 10^{ - 14}$ A/μm, and *I*_ON_/*I*_OFF_ = $5.47 \times 10^{7}$, and *SS*_avg_ = 24.48 mV/decade. Furthermore, using Si_0.7_Ge_0.3_ and Si as the source and drain materials, respectively. The *I*_ON_ and *I*_OFF_ characteristics have been further improved. Specifically, the resulting *I*_ON_ = 2.26 $\times 10^{ - 6}$ A/μm, *I*_OFF_ = $1.84 \times 10^{ - 15}$ A/μm, *I*_ON_/*I*_OFF_ = 1.22 $\times 10^{9}$, and SS_avg_ = 19.12 mV/decade. We believe that this device can be practically fabricated and possess excellent *SS* characteristics as well as *I*_ON_/*I*_OFF_. This would enable the device to maintain switching speed even under low-power-consumption applications.

## Data Availability

The data and analysis results generated in this study are available from the corresponding author on reasonable request.
